# Comparison of low molecular weight glutenin subunits identified by SDS-PAGE, 2-DE, MALDI-TOF-MS and PCR in common wheat

**DOI:** 10.1186/1471-2229-10-124

**Published:** 2010-06-24

**Authors:** Li Liu, Tatsuya M Ikeda, Gerard Branlard, Roberto J Peña, William J Rogers, Silvia E Lerner, María A Kolman, Xianchun Xia, Linhai Wang, Wujun Ma, Rudi Appels, Hisashi Yoshida, Aili Wang, Yueming Yan, Zhonghu He

**Affiliations:** 1 Institute of Crop Science, National Wheat Improvement Center/The National Key Facility for Crop Genetic Resources and Genetic Improvement, Chinese Academy of Agricultural Sciences (CAAS), 12 Zhongguancun South Street, Beijing 100081, China; 2 National Agriculture and Food Research Organization, 6-12-1 Nishifukatsu, Fukuyama, Hiroshima, 721-8514, Japan; 3 INRA Station d'Amelioration des Plantes, Domaine de Crouelle, 63039 Clermont- Ferrand, France; 4 CIMMYT Mexico, Apdo, Postal, 6-641, 06600, Mexico, DF, Mexico; 5 CIISAS, CICPBA-BIOLAB AZUL, Facultad de Agronomía, Universidad Nacional del Centro de la Provincia de Buenos Aires, Av. República Italia 780, C.C. 47, (7300), Azul, Provincia de Buenos Aires, Argentina. CONICET INBA -CEBB-MdP; 6 CRESCAA, Facultad de Agronomía, Universidad Nacional del Centro de la Provincia de Buenos Aires, Av. República Italia 780, C.C. 47, (7300), Azul, Provincia de Buenos Aires, Argentina; 7 Western Australia Department of Agriculture and Food, State Agriculture Biotechnology Center, Murdoch University, Murdoch, WA 6150, Australia; 8 National Agriculture and Food Research Organization, 3-1-1 Kannondai, Tsukuba, Ibaraki, 305-8517, Japan; 9 Key Laboratory of Genetics and Biotechnology, College of Life Science, Capital Normal University, 105 Xisanhuan Beilu, Beijing 100037, China; 10 International Maize and Wheat Improvement Center (CIMMYT) China Office, c/o CAAS, 12 Zhongguancun South Street, Beijing 100081, China

## Abstract

**Background:**

Low-molecular-weight glutenin subunits (LMW-GS) play a crucial role in determining end-use quality of common wheat by influencing the viscoelastic properties of dough. Four different methods - sodium dodecyl sulfate polyacrylamide gel electrophoresis (SDS-PAGE), two-dimensional gel electrophoresis (2-DE, IEF × SDS-PAGE), matrix-assisted laser desorption/ionization time-of-flight mass spectrometry (MALDI-TOF-MS) and polymerase chain reaction (PCR), were used to characterize the LMW-GS composition in 103 cultivars from 12 countries.

**Results:**

At the *Glu-A3 *locus, all seven alleles could be reliably identified by 2-DE and PCR. However, the alleles *Glu-A3e *and *Glu-A3d *could not be routinely distinguished from *Glu-A3f *and *Glu-A3g*, respectively, based on SDS-PAGE, and the allele *Glu-A3a *could not be differentiated from *Glu-A3c *by MALDI-TOF-MS. At the *Glu-B3 *locus, alleles *Glu-B3a*, *Glu-B3b*, *Glu-B3c*, *Glu-B3g*, *Glu-B3h *and *Glu-B3j *could be clearly identified by all four methods, whereas *Glu-B3ab*, *Glu-B3ac*, *Glu-B3ad *could only be identified by the 2-DE method. At the *Glu-D3 *locus, allelic identification was problematic for the electrophoresis based methods and PCR. MALDI-TOF-MS has the potential to reliably identify the *Glu-D3 *alleles.

**Conclusions:**

PCR is the simplest, most accurate, lowest cost, and therefore recommended method for identification of *Glu-A3 *and *Glu-B3 *alleles in breeding programs. A combination of methods was required to identify certain alleles, and would be especially useful when characterizing new alleles. A standard set of 30 cultivars for use in future studies was chosen to represent all LMW-GS allelic variants in the collection. Among them, Chinese Spring, Opata 85, Seri 82 and Pavon 76 were recommended as a core set for use in SDS-PAGE gels. *Glu-D3c *and *Glu-D3e *are the same allele. Two new alleles, namely, *Glu-D3m *in cultivar Darius, and *Glu-D3n *in Fengmai 27, were identified by 2-DE. Utilization of the suggested standard cultivar set, seed of which is available from the CIMMYT and INRA Clermont-Ferrand germplasm collections, should also promote information sharing in the identification of individual LMW-GS and thus provide useful information for quality improvement in common wheat.

## Background

Glutenin proteins are the major factors responsible for the unique viscoelastic characteristics of wheat dough. They determine rheological properties and bread-making performance [1-3]. The polymeric glutenin proteins, with molecular weights ranging from less than 300 to more than 1,000 kDa, are composed of two groups of subunits. These subunits include the LMW-GS, which are similar in size and structure to the *γ*- gliadins (30-40 kDa), and the high-molecular-weight glutenin subunits (HMW-GS) which range in molecular mass from ~65 to 90 kDa [4]. The LMW-GS represent about one-third of the total seed protein and ~60% of total glutenins [5], and are essential in determining dough properties, such as dough extensibility [6] and gluten strength [2]. Hence characterization of allelic variation among cultivars and investigation of their relationships with end-use quality has been a key area of research on quality improvement during the last 15 years, and is the basis for the success of using specific LMW-GS alleles in breeding programs [7-9].

The genes coding for LMW-GS are located on the short arms of homoeologous group 1 chromosomes at the *Glu-A3*, *Glu-B3 *and *Glu-D3 *loci, and are tightly linked to the *Gli-1 *loci [10-12]. The *Glu-A3 *locus on chromosome 1A encodes relatively few LMW-GS, with alleles *Glu-A3e *in hexaploid or common wheat and *Glu-A3h *in tetraploid wheat being null alleles that do not express any *Glu-A3 *product [13,14]. In contrast, there is extensive variation for LMW-GS encoded by chromosome 1B in common wheat. The *Glu-D3 *locus has less variability with five alleles reported originally by Gupta and Shepherd [13], four alleles by Lerner et al. [15] and only three alleles observed by Jackson et al. [16] and Eagles et al. [17]. Nonetheless, recent studies using protein and PCR analyses have identified 11 *Glu-D3 *alleles [18,19], suggesting that a reexamination should be carried out to clarify the genetic variability at this locus.

Despite the abundance of the LMW-GS, they have received much less attention than the HMW-GS, probably due to their complexity, heterogeneity and co-migration with gliadins in SDS-PAGE [19,20]. In the SDS-PAGE system, utilizing gliadins as indicators provided an indirect way to define LMW-GS alleles [16]. The 2-DE analytical process that could generate much more information than SDS-PAGE [21] was not generally recommended for use in breeding programs, due to its time-consuming procedure, high costs and skill requirements. MALDI-TOF-MS is currently the most efficient method to analyze proteins and requires only 4-5 minutes per sample. It is a high throughput technology for analyzing wheat gluten proteins [22-25], but being relatively new and expensive, few wheat breeding programs can afford to acquire such equipment. Recently, a simple, rapid and sensitive PCR approach, has proven to be a very useful tool for identifying LMW-GS composition in common wheat [19,26-28].

LMW-GS were first identified by gel filtration and starch gel electrophoresis of extracts of wheat flour [29,30]. They were classically subdivided into B, C, and D groups (no relationship to the A, B and D genomes of wheat), according to their electrophoretic mobilities in SDS-PAGE and their isoelectric points (pI) [31]. Based on the locations of cysteine residues involved in the formation of intermolecular disulfide bridges, Ikeda et al. [32] classified LMW gene sequences into six types, each containing several different groups based upon differences in their N- and C-terminal acid-amino compositions. Altogether, 12 groups were differentiated, but an additional five groups were reported by Juhász and Gianibelli [33].

The allelic nomenclature system for the LMW-GS was defined through the chromosomal location of the DNA coding regions by Gupta and Shepherd [13] and was reviewed by Jackson et al. [16]. Branlard et al. [34] proposed a schematic presentation of SDS-PAGE relative subunit mobilities to characterize the different alleles encoded at *Glu-A3*, *Glu-B3 *and *Glu-D3 *loci. Ikeda et al. [35] recently compared *Glu-3 *allele identifications from five laboratories, confirming inconsistencies between laboratories in identifying *Glu-3 *alleles due to differences between the separation and identification methods. The study also indicated new *Glu-3 *alleles in a number of the cultivars analyzed.

The N-terminal sequences of LMW-GS were used to divide the protein subunits into two main groups [32,36]. The first group corresponded to typical LMW-GS, i.e., LMW-i (or i-type, first amino acid isoleucine) and LMW-m (or m-type, methionine) types, and the second group, named gliadin-like sequences [37] as these subunits have N-terminal sequences similar to *α*-, *γ*- and *ω*- gliadins. Most gliadins are monomeric, but some have an extra cys that allows them to be incorporated into glutenin polymers. Payne [1] termed the prominent bands observed by SDS-PAGE of reduced glutenin protein as A (HMW-GS), B (many of the LMW-GS) and C (the smaller LMW-GS). Later, other researchers also observed larger gliadin-like subunits, between the A and B bands, and they named them as D- subunits [31]. Most of the B- subunits were shown to possess i-, m- or s (serine) -type N-terminal sequences [38]. C- subunits including *α*-, and *γ*- gliadins-like subunits as well as subunits with classic LMW-GS sequences occur in large numbers, although their relative amounts are lower than those of B- subunits. Similarly, D- subunits have N-terminal sequences that correspond to *ω*- gliadins, another type of gliadin-like sequence [2,39,40].

The use of two distinct nomenclature systems, one based upon the relative mobilities in SDS-PAGE and the other upon N-terminal sequences, make it extremely difficult to compare work from different laboratories. The main ambiguities from these different classification systems can be summarized as follows: 1) at the *Glu-A3 *locus, both *Glu-A3a *and *Glu-A3c *were reported for the same cultivar, and similarly, *Glu-A3a*, *Glu-A3b*, *Glu-A3c*, *Glu-A3d *were reported to be identical to *Glu-A3e*; 2) at the *Glu-B3 *locus, results differed for *Glu-B3b *and *Glu-B3g*, and for *Glu-B3f *and *Glu-B3g *in the same cultivars; and 3) at the *Glu-D3 *locus, there was ambiguity between *Glu-D3a *and *Glu-D3c*, and between *Glu-D3a *and *Glu-D3b *in the same cultivars [41]. As a consequence of these problems, reports of correlations between certain allelic forms of LMW-GS and quality parameters in common wheat have often been contradictory [7,42-45]. It is, therefore, essential to establish a simple and uniform classification through a set of standard cultivars for each LMW-GS allele.

In 2005, a cooperative program was developed among the following five laboratories to establish such a set of standard cultivars for identifying LMW-GS alleles: Chinese Academy of Agricultural Sciences (CAAS, China), International Maize and Wheat Improvement Center (CIMMYT, Mexico), National Institute for Agricultural Research (INRA, France), National Agriculture and Food Research Organization (NARO, Japan), and National University of the Center of the Province of Buenos Aires (Universidad Nacional, Argentina). A set of 103 cultivars used in various previously studies [35] in 12 countries was assembled and distributed to all laboratories, including Murdoch University as an additional laboratory, for the identification of LMW-GS alleles. Their preliminary *Glu-3 *allelic assignments were summarized in a previous paper [35]. The objectives of the current paper are 1) to compare the LMW-GS compositions obtained by SDS-PAGE, 2-DE, MALDI-TOF-MS and PCR in order to clearly identify the protein compositions of cultivars in the collection; and 2) to establish a set of standard cultivars for the identification of LMW-GS alleles, enabling information regarding the effects of individual LMW-GS on gluten properties to be readily and continuously shared between laboratories and applied in breeding programs.

## Results and discussion

### Analysis of LMW-GS by SDS-PAGE

**Figure 1:**
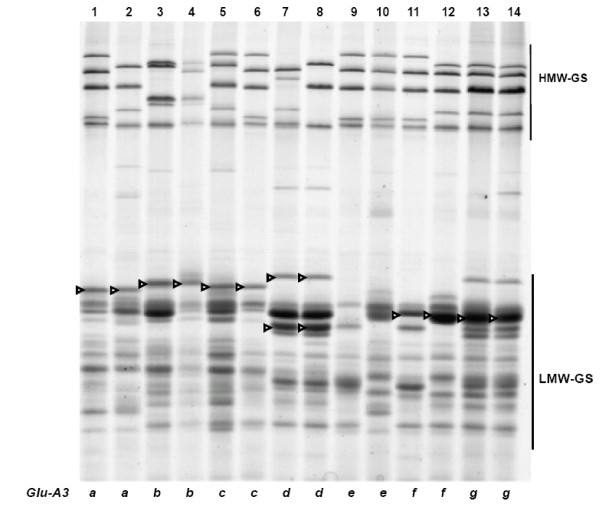
**SDS-PAGE of LMW-GS**. The LMW-GS are propanol-insoluble fractions extracted with 50% propanol + 1% w/v DTT + 1.4% v/v 4-vinylpyridine (The same as below). Cultivars: 1. Neixiang 188, 2. Chinese Spring, 3. Gabo, 4. Pavon 76, 5. Pitic, 6. Seri, 7. Nidera Baguette 10, 8. Cappelle-Desprez, 9. Amadina, 10. Marquis, 11. Kitanokaori, 12. Renan, 13. Bluesky, 14. Glenlea. Arrow heads indicate bands corresponding to different *Glu-A3 *alleles.

**Figure 2:**
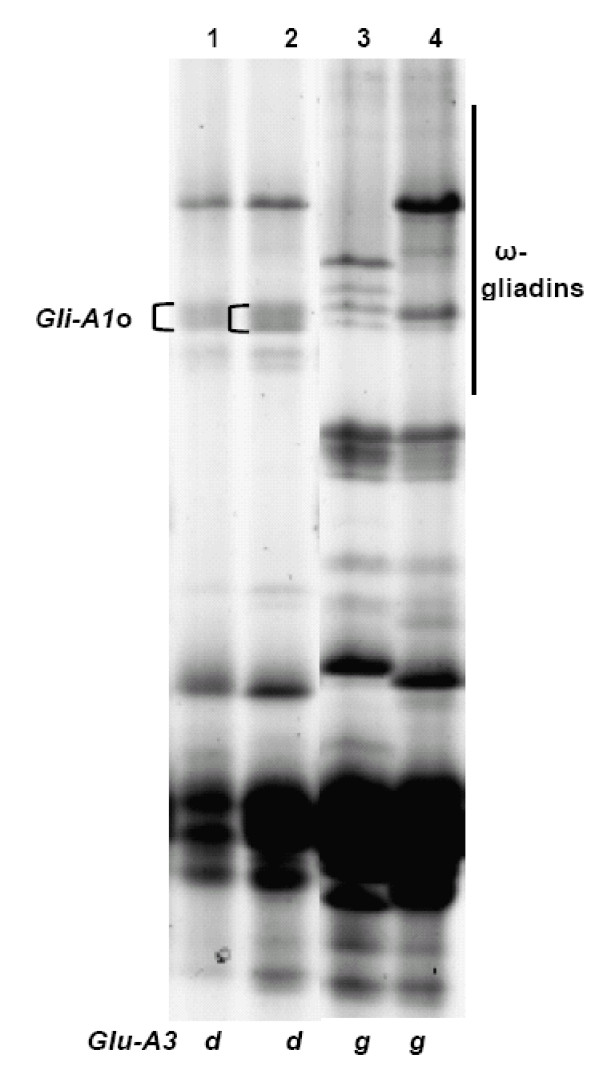
**SDS-PAGE of gliadins**. The gliadins are 50% propanol (v/v) soluble fractions (The same as below). Cultivars in lanes 7, 8, 13, and 14, correspond to the same shown with same number in Figure 1. The linkage between *Gli-A1o *(indicated in lanes 7 and 8 in the omega-gliadin zone) and *Glu-A3d *helps to differentiate the latter from *Glu-A3g*.

The LMW-GS compositions identified in participating laboratories by SDS-PAGE were combined and listed in Table 1 (details available upon request); discrepancies among different laboratories were discussed by Ikeda et al. [35]. At the *Glu-A3 *locus, alleles *Glu-A3a*, *Glu-A3b*, *Glu-A3c *and *Glu-A3f *could be readily identified using SDS-PAGE (Figure 1). Alleles *Glu-A3d *and *Glu-A3g *could be differentiated with the aid of the gliadin SDS-PAGE gel; by the presence or absence of the *Gli-A1o *allele, which we believe is linked to *Glu-A3d*, but not to *Glu-A3g *(Figure 2). It was difficult to distinguish *Glu-A3f *from *Glu-A3e *(null allele). In previous studies [7,46,47] both alleles tended to be detected as *Glu-A3e*.

**Table 1:** Compositions of LMW-GS alleles in 103 wheat cultivars identified by SDS-PAGE, 2-DE, MALDI-TOF-MS and allele-specific markers

Cultivar	Origin	*Glu-A3*	*Glu-B3*	*Glu-D3*
Aca 303	Argentina	f/f/f/f*	h/h/h/h	c/c/c/-
Aca 601	Argentina	f/f/f/f	b/b/b/b	c/c/c/-
Aca 801	Argentina	c/c/a or c/c	g/ac/g/g	b/b/b/-
Buck Brasil	Argentina	f/f/f/f	g/ac/g/g	d/d/?/-
Buck Mejorpán	Argentina	f/f/f/f	b/b/b/b	c/c/c/-
Buck Pingo	Argentina	f/f/f/f	i/ad/d or i/i	c/c/c/-
Klein Capricornio	Argentina	c/c/a or c/c	h/h/h/h	b/b/b/-
Klein Chaja	Argentina	c/c/a or c/c	h/h/h/h	b/b/b/-
Klein Flecha	Argentina	c/c/a or c/c	h/h/h/h	b/b/b/-
Klein Jabal 1	Argentina	d/d/d/g	g/g/g/g	c/c/c/-
Klein Martillo	Argentina	e/e/e/e	j/j/j/j	b/b/b/-
Klein Proteo	Argentina	g/g/e/g	g/ac/g/g	b/b/b/-
Nidera Baguette 10	Argentina	d/d/d/d	g/g/g/g	c/c/c/-
Nidera Baguette 20	Argentina	f/f/f/f	g/g/g/g	c/c/c/-
ProINTA Amanecer	Argentina	f/f/f/f	j/j/j/j	a/a/b/-
ProINTA Colibr 1	Argentina	d/d/d/d	b/b/b/b	a/a/a/-
ProINTA Isla Verde	Argentina	b/b/b/b	b/b/b/b	b/b/b/-
ProINTA Redomon	Argentina	c/c/a or c/c	h/h/h/h	c/c/c/-
Thomas Nevado	Argentina	c/c/a or c/c	j/j/j/j	b/b/b/-
Angas	Australia	c/c/a or c/c	g/g/g/g	c/c/c/-
Avocet	Australia	c/c/a or c/c	b/b/b/b	b/b/b/-
Carnamah	Australia	c/c/a or c/c	i/ad/d or i/i	c/c/c/-
Gabo	Australia	b/b/b/b	b/b/b/b	b/b/b/-
Grebe	Australia	c/c/a or c/c	j/j/j/j	b/b/b/-
Halberd	Australia	e/e/e/e	c/c/c/c	c/c/c/-
Insignia	Australia	f/f/f/f	c/c/c/c	c/c/c/-
Millewa	Australia	c/c/a or c/c	g/g/g/g	b/b/b/-
Spear	Australia	e/e/e/e	h/h/h/h	c/c/c/-
Stiletto	Australia	c/c/a or c/c	h/h/h/h	c/c/c/-
Tasman	Australia	b/b/b/b	i/ad/d or i/i	a/a/a/-
Trident	Australia	e/e/e/e	h/h/h/h	c/c/c/-
Westonia	Australia	c/c/a or c/c	h/h/h/h	c/c/c/-
Wilgoyne	Australia	d/d/d/d	h/h/h/h	b/b/b/-
AC Vista	Canada	e/e/e/e	i/ad/d or i/i	c/c/c/-
Bluesky	Canada	g/g/e/g	g/g/g/g	c/c/c/-
Glenlea	Canada	g/g/e/g	g/g/g/g	c/c/c/-
Katepwa	Canada	e/e/e/e	h/h/h/h	c/c/c/-
Marquis	Canada	e/e/e/e	b/b/b/b	a/a/a/-
Neepawa	Canada	e/e/e/e	h/h/h/h	c/c/c/-
Pioneer	Canada	e/e/e/e	i/ad/d or i/i	c/c/c/-
99G46	China	f/f/f/f	j/j/j/j	c/c/c/-
CA9641	China	d/d/d/d	h/h/h/h	c/c/c/-
CA9722	China	c/c/a or c/c	h/h/h/h	c/l/c/-
Chinese Spring	China	a/a/a or c/a	a/a/a/a	a/a/a/-
Demai 3	China	c/c/a or c/c	i/d or i/d or i/i	b/b/b/-
Fengmai 27	China	c/c/a or c/c	f/f/f/f	a/n/a/-
Guanfeng 2	China	c/c/a or c/c	b/b/b/b	a/a/a/-
Huaimai 16	China	f/f/f/f	h/h/h/h	c/c/c/-
Jing 411	China	c/c/a or c/c	h/h/h/h	c/l/c/-
Lumai 23	China	c/c/a or c/c	d/d or i/d or i/d	c/l/c/-
Neixiang 188	China	a/a/a or c/a	j/j/j/j	a/a/a/-
Shan 229	China	c/c/a or c/c	j/j/j/j	b/b/b/-
Wanmai 33	China	d/d/d/d	g/g/g/g	a/a/b/-
Yan 239	China	c/c/a or c/c	j/j/j/j	b/b/b/-
Yangmai 158	China	c/c/a or c/c	g/g/g/g	c/c/c/-
Yumai 54	China	c/c/a or c/c	d/d or i/d or i/d	c/c/c/-
Yumai 63	China	c/c/a or c/c	d/d or i/d or i/d	c/c/c/-
Yumai 69	China	c/c/a or c/c	d/d or i/d or i/d	a/a/b/-
Zhongyou 9507	China	d/d/d/d	b/b/b/b	c/c/c/-
Zhongyou 9701	China	d/d/d/d	d/d or i/d or i/d	c/c/c/-
Zhongyu 415	China	c/c/a or c/c	d/d or i/d/d	c/c/c/-
Ruso	Finland	c/c/a or c/c	i/ad/d or i/i	a/a/a/-
Brimstone	France	c/c/a or c/c	g/g/g/g	d/d/?/-
Cappelle-Desprez	France	d/d/d/d	g/g/g/g	c/c/c/-
Chopin	France	c/c/a or c/c	h/h/h/h	c/c/c/-
Clément	France	f/f/f/f	j/j/j/j	c/c/c/-
Courtot	France	c/c/a or c/c	b/b/b/b	c/l/c/-
Darius	France	d/d/d/d	g/g/g/g	b/m/m/-
Etoile de Choisy	France	d/d/d/d	i/d or i/d or i/i	c/l/c/-
Festin	France	f/f/f/f	b/b/b/b	c/l/c/-
Magali Blondeau	France	e/e/e/e	g/g/g/f	b/b/b/-
Magdalena	France	d/d/d/d	b/b/b/b	a/a/a/-
Petrel	France	d/d/d/d	h/h/h/h	c/c/c/-
Renan	France	f/f/f/f	b/b/b/b	b/b/b/-
Soissons	France	c/c/a or c/c	b/b/b/b	c/c/c/-
Thesee	France	c/c/a or c/c	g/ac/g/g	c/l/c/-
Apollo	Germany	d/d/d/d	j/j/j/j	c/c/c/-
Manital	Italy	c/c/a or c/c	b/b/b/b	a/a/a/-
Salmone	Italy	c/c/a or c/c	c/c/c/g	c/c/c/-
Aoba-komugi	Japan	e/e/e/e	b/b/b/b	c/c/c/-
Eshimashinriki	Japan	c/c/a or c/c	d/d or i/d or i/d	a/a/a/-
Haruyutaka	Japan	c/c/a or c/c	h/h/h/h	b/b/b/-
Kanto 107	Japan	c/c/a or c/c	g/g/g/g	a/a/a/-
Kitanokaori	Japan	f/f/f/f	j/j/j/j	c/c/c/-
Nanbu-komugi	Japan	d/d/d/d	b/ab/b/b	a/a/a/-
Norin 61	Japan	d/d/d/d	i/d or i/d or i/-	c/c/c/-
Norin 67	Japan	c/c/a or c/c	g/g/g/g	b/b/b/-
Shinchunaga	Japan	c/c/a or c/c	i/ad/d or i/-	a/a/a/-
Shirane-komugi	Japan	e/e/e/e	i/ad/d or i/i	a/a/a/-
Amadina	Mexico	e/e/e/e	j/j/j/j	c/l/c/-
Attila	Mexico	c/c/a or c/c	h/h/h/h	b/b/b/-
Heilo	Mexico	f/f/f/f	i/ad/d or i/i	c/l/c/-
Opata 85	Mexico	b/b/b/b	i/ad/d or i/i	a/a/a/-
Pastor	Mexico	c/c/a or c/c	g/g/g/g	b/b/b/-
Pavon 76	Mexico	b/b/b/b	h/h/h/h	b/b/b/-
Pitic	Mexico	c/c/a or c/c	b/b/b/b	b/b/b/-
Rebeca	Mexico	c/c/a or c/c	g/g/g/g	b/b/b/-
Seri 82	Mexico	c/c/a or c/c	j/j/j/j	b/b/b/-
Orca	Netherlands	d/d/d/d	d/d or i/d or i/d	c/c/c/-
Pepital	Netherlands	f/f/f/f	d/d or i/d or i/d	c/l/c/-
Ernest	USA	d/d/d/d	d/d or i/d or i/d	d/?/?-
Splendor	USA	e/e/e/e	g/g/g/g	b/b/b/-
Verde	USA	f/f/f/f	h/h/h/h	c/c/c/-

*, the first, second, third and fourth symbol in each column are alleles of *Glu-3 *loci identified by SDS-PAGE, 2-DE, MALDI-TOF-MS and PCR, respectively.

Data from five laboratories are combined data for SDS-PAGE. - indicates data not available

**Figure 3:**
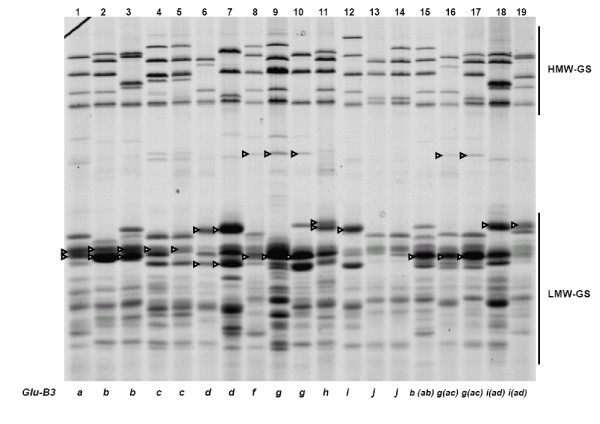
**SDS-PAGE of LMW-GS**. Cultivars: 1. Chinese Spring, 2. Renan, 3. Gabo, 4. Insignia, 5. Halberd, 6. Pepital, 7. Ernest, 8. Fengmai 27, 9. Splendor, 10. Cappelle-Desprez, 11. Aca 303, 12. Norin 61, 13. Grebe, 14. Seri 82, 15. Nanbu-komugi, 16. Thesee, 17. Aca 801, 18. Heilo, 19. Opata. Arrow heads indicate bands corresponding to different *Glu-B3 *alleles. *Glu-B3 *allele designation between brackets for cultivars in lanes 15-19 correspond to provisional nomenclature as indicated by spot differences in 2-DE.

**Figure 4:**
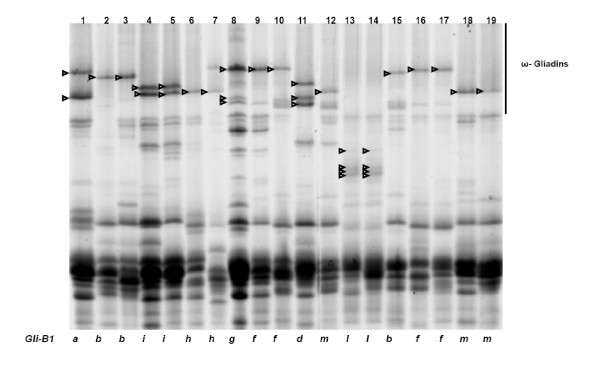
**SDS-PAGE of gliadins**. Cultivars in lanes 1-19 are the same as in Figure 3. Arrow heads indicate bands corresponding to different *Gli-B1 *alleles. *Glu-B3 *and *Gli-B1 *alleles in each of the lanes 1 to 19 of Figures 3 and 4 are tightly linked. The bands indicated with arrow heads of Figure 4 are used as assisted bands for the identification of some *Glu-B3 *alleles shown on Figure 3 based on *Glu-B3*/*Gli-B1 *linkage.

Figure 3 shows cultivars representing different *Glu-B3 *alleles. At the *Glu-B3 *locus, three alleles, *Glu-B3d*, *Glu-B3h *and *Glu-B3i*, each carried the slowest LMW-GS bands in the SDS-PAGE region B among the cultivars studied. The slowest *Glu-B3 *band, *Glu-B3b*, almost coincided with *Glu-A3a*, but the *Glu-B3b *band was usually lighter and thinner, permitting their discrimination. Allele *Glu-B3f *could not be reliably discriminated from *Glu-B3g *since these bands had very similar mobilities, including the presence of a band in the SDS-PAGE region (Figure 3, lanes 8-10) as previously reported [7,34,41]. However, taking advantage of the *Glu-B3*/*Gli-B1 *linkage, one can look at the omega-gliadins region in SDS-PAGE, to identify with confidence several of the *Glu-B3 *alleles (Figure 4). Actually, differentiating between several *Glu-B3 *alleles is possible only looking at both, gliadin and glutenin SDS-PAGE gels. Using this criteria, *Glu-B3 *alleles in lanes 15, 16, 17, 18, and 19, seem to correspond to *Glu-B3b*, *Glu-B3g*, *Glu-B3g*, *Glu-B3i*, and *Glu-B3i*, respectively (Figures 3 and 4), however, 2-DE analysis indicates that these genotypes correspond to new alleles provisionally designated as *Glu-B3ab*, *Glu-B3ac*, *Glu-B3ac*, *Glu-B3ad*, and *Glu-B3ad*, respectively (Figure 3).

**Figure 5:**
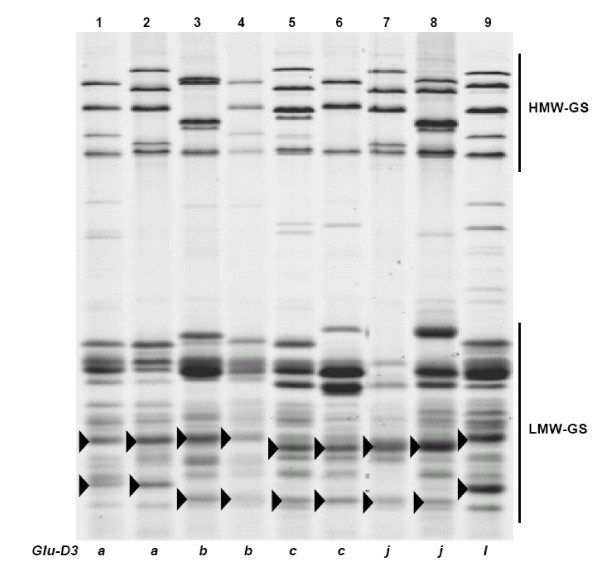
**SDS-PAGE of LMW-GS**. Cultivars: 1. Chinese Spring, 2. Neixiang 188, 3. Gabo, 4. Avocet, 5. Insignia, 6. Cappelle- Desprez, 7. Amadina, 8. Heilo, 9. Fengmai 27. Arrow heads indicate bands corresponding to different *Glu-D3 *alleles.

Figure 5 shows cultivars representing different *Glu-D3 *alleles. Although alleles *Glu-D3a*, *Glu-D3b*, *Glu-D3c *and *Glu-D3d *were frequently identified in germplasm from various origins [35], only alleles *Glu-D3a*, *Glu-D3b *and *Glu-D3d *were consistently differentiated [34]. *Glu-D3 *alleles had similar mobilities to gliadins and were generally faintly stained due to the rapid diffusion of low molecular mass proteins from the gel. Thus the identification of *Glu-D3 *alleles was quite difficult using only SDS-PAGE, leading to the reported discrepancies [13,19,41]. Although improvements to the SDS-PAGE protocol now allow differentiating several of the *Glu-D3 *alleles with more certainty, as it is shown in Figure 5, other methods for definitive identification of these alleles, such as 2-DE, MALDI-TOF-MS and PCR, had to be implemented to facilitate identification of *Glu-D3 *alleles.

### Characterization of LMW-GS by 2-DE

**Figure 6:**
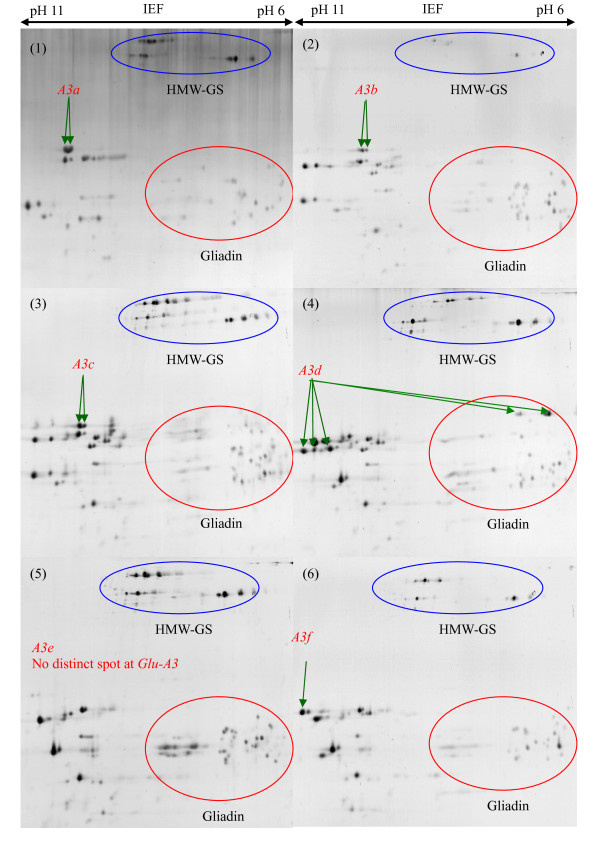
**Identification of LMW-GS by two-dimensional gel electrophoresis (2-DE)**. Discrimination of alleles *Glu-A3a*, *Glu-A3b*, *Glu-A3c*, *Glu-A3d*, *Glu-A3e *and *Glu-A3f*. Cultivars: 1. Neixiang 188, 2. Gabo, 3. Pitic, 4. Nidera Baguette 10, 5. Amadina, 6. Kitanokaori.

**Figure 7:**
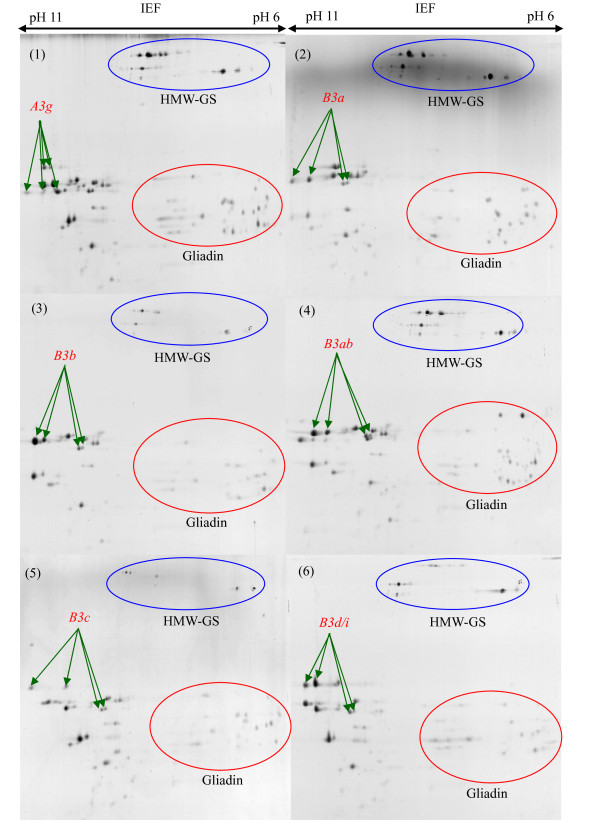
**Identification of LMW-GS by two-dimensional gel electrophoresis (2-DE)**. Discrimination of alleles *Glu-A3g*, *Glu-B3a*, *Glu-B3b*, *Glu-B3ab*, *Glu-B3c *and *Glu-B3d/i*. Cultivars: 1. Bluesky, 2. Chinese Spring, 3. Renan, 4. Nanbu-komugi, 5. Insignia, 6. Pepital. Letters preceding and following "/" indicate pairs of alleles that could not be reliably distinguished.

**Figure 8:**
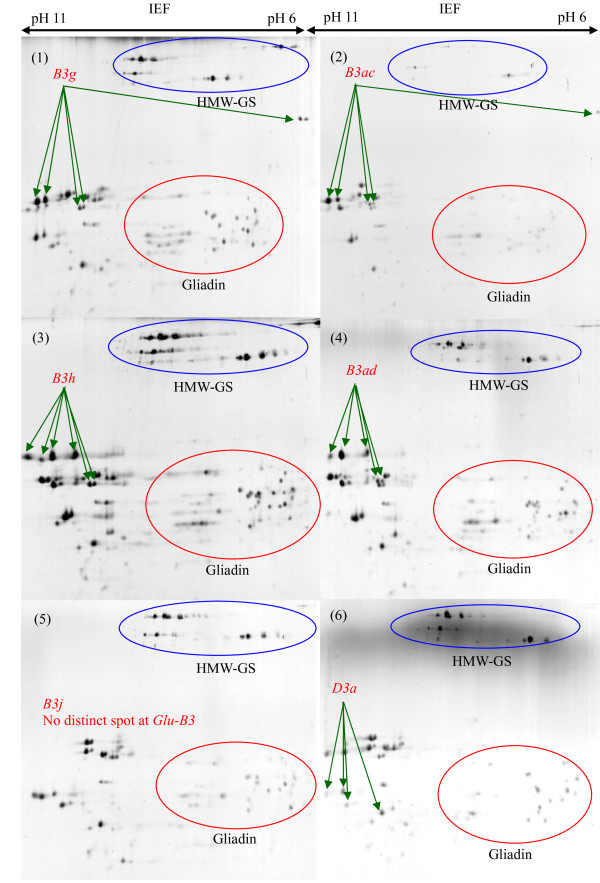
**Identification of LMW-GS by two-dimensional gel electrophoresis (2-DE)**. Discrimination of alleles *Glu-B3g*, *Glu-B3ac*, *Glu-B3h*, *Glu-B3ad*, *Glu-B3j *and *Glu-D3a*. Cultivars: 1. Splendor, 2. Thesee, 3. Aca 303, 4. Heilo, 5. Grebe, 6. Chinese Spring.

**Figure 9:**
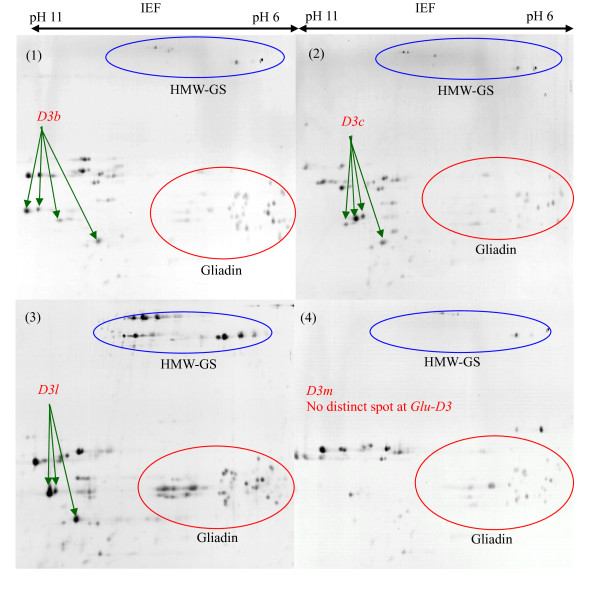
**Identification of LMW-GS by two-dimensional gel electrophoresis (2-DE)**. Discrimination of alleles *Glu-D3b*, *Glu-D3c*, *Glu-D3l *and *Glu-D3m*. Cultivars: 1. Gabo, 2. Insignia, 3. Amadina, 4. Darus.

The identification of the LMW-GS alleles by 2-DE was consistent between the two laboratories. The discrimination between LMW-GS alleles in the collection by high resolution 2-DE is illustrated in Figures 6, 7, 8 and 9 and the results are shown in Table 1. The *Glu-A3 *alleles *Glu-A3d *(Figure 6, (4)), *Glu-A3e *(Figure 6, (5)), *Glu-A3f *(Figure 6, (6)) and *Glu-A3g *(Figure 7, (1)), were readily differentiated on the basis of protein spots with clearly different molecular masses and pI. Alleles *Glu-A3a *(Figure 6, (1)), *Glu-A3b *(Figure 6, (2)) and *Glu-A3c *(Figure 6, (3)) had identical pI but different molecular masses, making it possible to discriminate between them.

At the *Glu-B3*, the alleles *Glu-B3ab *(Figure 7, (4)), *Glu-B3ac *(Figure 8, (2)), *Glu-B3h *(Figure 8, (3)), *Glu-B3ad *(Figure 8, (4)) and *Glu-B3j *(Figure 8, (5)) were easily differentiated by protein spots having different molecular masses and pI. Alleles *Glu-B3ab *(Figure 7, (4)), *Glu-B3ac *(Figure 8, (2)) and *Glu-B3ad *(Figure 8, (4)) were each discriminated from *Glu-B3b *(Figure 7, (3)), *Glu-B3g *(Figure 8, (1)) and *Glu-B3i *(image not provided) by two distinct protein spots. Although the majority of the protein spots for alleles *Glu-B3b *and *Glu-B3g *had identical molecular masses and pI, they could be discriminated since allele *Glu-B3g *had one additional spot, at pH6, located between the HMW-GS and gliadins. There were no obvious differences in molecular mass or pI between alleles *Glu-B3d *(Figure 7, (6)) and *Glu-B3i *(image not provided), or between *Glu-B3f *(image not provided) and *Glu-B3g *(Figure 8, (1)), making differentiation by 2-DE impossible.

At the *Glu-D3*, only *Glu-D3c *(Figure 9, (2)), *Glu-D3l *(Figure 9, (3)) and *Glu-D3m *(Figure 9, (4)) could be definitely identified by 2-DE. Allele *Glu-D3l *(Figure 9, (3)) had two more distinctive spots compared to *Glu-D3c *(Figure 9, (2)) in 2-DE separations. As expected, alleles *Glu-D3c *and *Glu-D3e *(image not provided) could not be separated by 2-DE. These alleles appeared to be the same based on SDS-PAGE and MALDI-TOF-MS in the present study as they were in a previous study [16].

2-DE did not distinguish *Glu-D3a *(Figure 8, (6)), *Glu-D3b *(Figure 9, (1)) and *Glu-D3d *(image not provided), hence further investigation should target discrimination of *Glu-D3 *alleles by combining 2-DE with other methods such as PCR.

### Identification of LMW-GS by MALDI-TOF-MS

**Figure 10:**
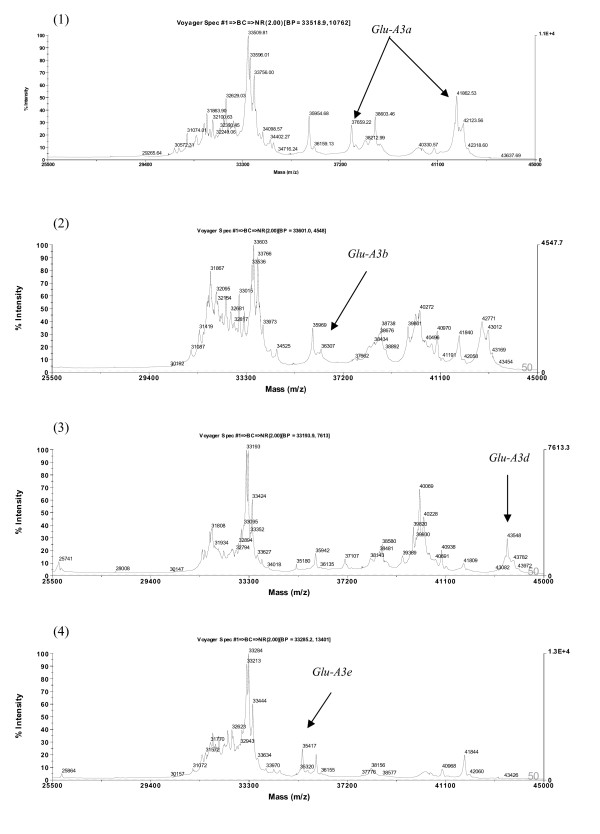
**Detection of LMW-GS by MALDI-TOF-MS**. Identification of alleles *Glu-A3a*, *Glu-A3b*, *Glu-A3d *and *Glu-A3e*. Cultivars: 1. Neixiang 188, 2. Gabo, 3. Nidera Baguette 10, 4. Amadina.

**Figure 11:**
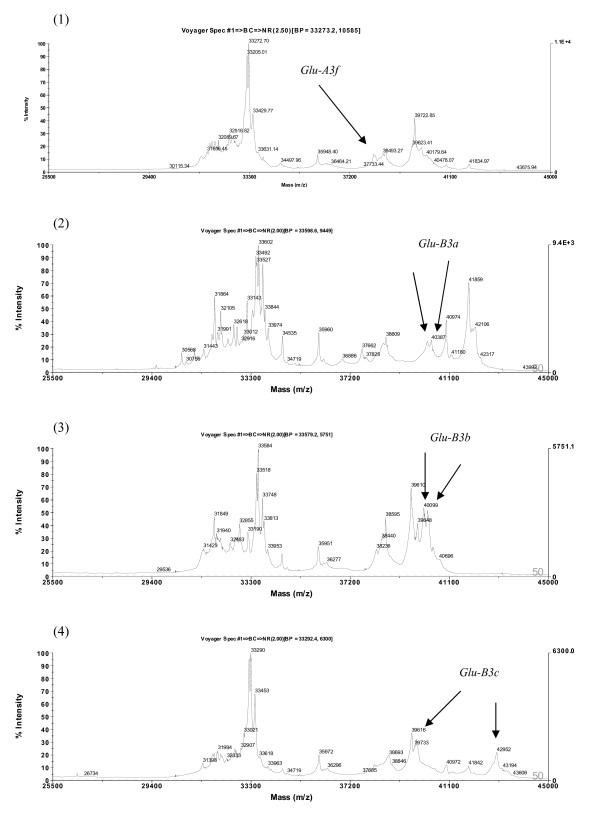
**Detection of LMW-GS by MALDI-TOF-MS**. Identification of alleles *Glu-A3f*, *Glu-B3a*, *Glu-B3b *and *Glu-B3c*. Cultivars: 1. Kitanokaori, 2. Chinese Spring, 3. Renan, 4. Insignia.

**Figure 12:**
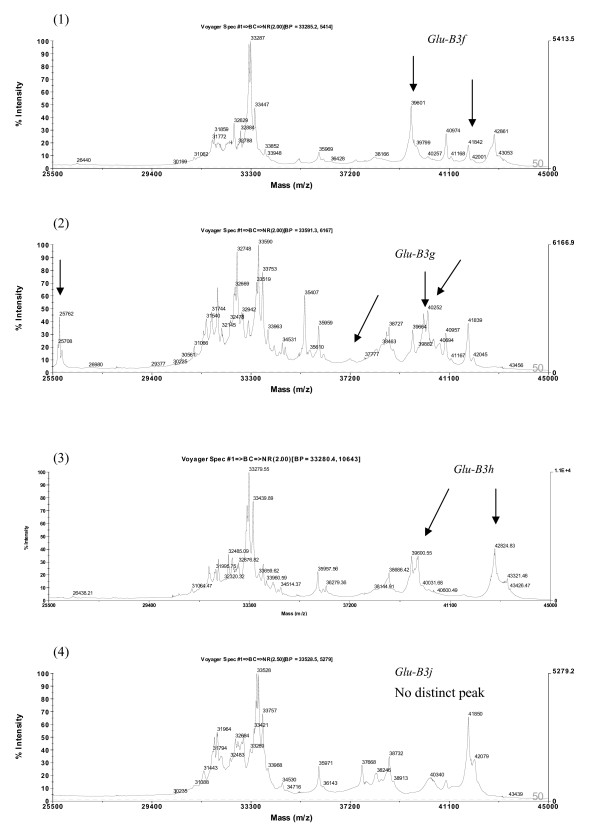
**Detection of LMW-GS by MALDI-TOF-MS**. Identification of alleles *Glu-B3f*, *Glu-B3g*, *Glu-B3h *and *Glu-B3j*. Cultivars: 1. Pepital, 2. Splendor, 3. Aca 303, 4. Grebe.

**Figure 13:**
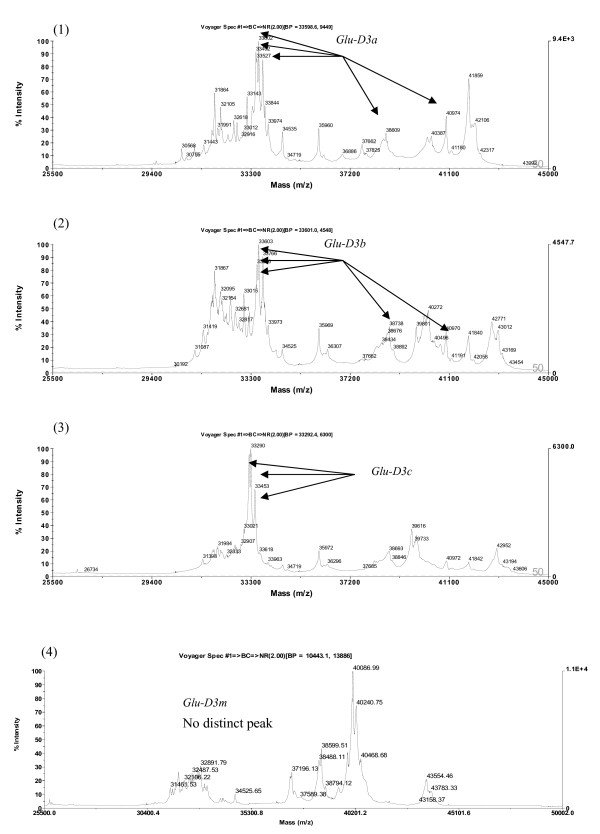
**Detection of LMW-GS by MALDI-TOF-MS**. Identification of alleles *Glu-D3a*, *Glu-D3b*, *Glu-D3c *and *Glu-D3m*. Cultivars: 1. Chinese Spring, 2. Gabo, 3. Insignia, 4. Darus.

The compositions of LMW-GS analyzed by MALDI-TOF-MS are presented in Table 1. As shown in Figures 10, 11, 12 and 13, the spectra of LMW subunits analyzed by this method consist of complex sets of peaks, consistent with the extensive diversity of the subunits. The LMW-GS exhibited molecular masses of 25-43 kDa in MALDI-TOF-MS spectra, considerably lower than the corresponding molecular masses of 42-51 kDa determined by SDS-PAGE and indicative of limitations of the SDS-PAGE method in determining the molecular masses of LMW glutenins [24]. Two major regions with masses from 30 to 35 kDa and from 36 to 43 kDa were separated in spectra of MALDI-TOF-MS (Figures 10, 11, 12 and 13). These regions correspond to the C- LMW-GS and B- LMW-GS classified by SDS-PAGE. The region with molecular masses of 30-35 kDa also corresponds in mass to the major gliadins range [1]. The results were in agreement with previous studies based on SDS-PAGE, where there were extensive overlaps between gliadins and LMW-GS with lower molecular masses [48].

MALDI-TOF-MS-based identification of LMW-GS alleles was established using a set of 19 near-isogenic lines (NIL) of cultivar Aroona (unpublished data, A Wang, W Ma, R Appels, Murdoch University, Australia). Most of the distinct peaks of the *Glu-A3 *alleles exhibited higher masses in the ranges of about 41.8-42.1 kDa and 43.5-43.8 kD, whereas the distinct peaks of the *Glu-D3 *alleles showed lower masses of 33.2-33.7 kDa. The middle masses in the ranges of about 40.1-40.2 kDa and 42.8-43.3 kDa corresponded to the *Glu-B3 *alleles. The distributions of distinct peaks of the *Glu-3 *alleles in the MALDI-TOF-MS were in agreement with their position in SDS-PAGE [34].

Compared to the other loci, *Glu-A3 *was less diverse and most protein bands had lower mobilities, so discrimination between them using SDS-PAGE is usually feasible. Similarly, most of the distinct peaks of the *Glu-A3 *alleles were well separated in MALDI-TOF-MS spectra, and alleles *Glu-A3b *(Figure 10, (2)), *Glu-A3d *(Figure 10, (3)), *Glu-A3e *(Figure 10, (4)) and *Glu-A3f *(Figure 11, (1)) were reliably discriminated.

The *Glu-B3 *alleles *Glu-B3a *(Figure 11, (2)), *Glu-B3b *(Figure 11, (3)), *Glu-B3c *(Figure 11, (4)), *Glu-B3h *(Figure 12, (3)), and *Glu-B3j *(Figure 12, (4)), as well as seven other alleles, were readily distinguished by MALDI-TOF-MS.

With regard the *Glu-D3 *locus, MALDI-TOF-MS clearly differentiated the *Glu-D3a *(Figure 13, (1)), *Glu-D3b *(Figure 13, (2)), *Glu-D3c *(Figure 13, (3)) and *Glu-D3m *(Figure 13, (4)) alleles. As expected, *Glu-D3e *(image not provided) could not be discriminated from *Glu-D3c *(Figure 13, (3)). Improved discrimination will be achieved as calibration technology improves. In addition, it may be of value to utilize the close linkage between gliadin and LMW glutenin alleles to further improve the power of MALDI-TOF-MS in differentiating LMW glutenin alleles.

### Detection of LMW-GS by allele specific PCR markers

Seven primer pairs [27], including *gluA3a*, *gluA3b*, *gluA3ac*, *gluA3d*, *gluA3e*, *gluA3f *and *gluA3g*, were used to identify *Glu-A3 *alleles (Table 1). The amplified fragment sizes for each marker were 529 bp for *Glu-A3a*, 894 bp for *Glu-A3b*, 967 bp for *Glu-A3d*, 158 bp for *Glu-A3e*, 552 bp for *Glu-A3f*, and 1345 bp for *Glu-A3g*, indicating that the *Glu-A3 *alleles in the collection could be readily distinguished from one another. Since no *Glu-A3c *allele-specific primer has been developed, identification of this allele required the use of the *gluA3ac *with a 573 bp band in combination with the marker *gluA3a *[27].

Ten primer pairs developed by Wang et al. [28] were utilized to test for *Glu-B3 *alleles and the results are summarized in Table 1. Specifically amplified fragments included 1095 bp for *Glu-B3a*, 1549 bp for *Glu-B3b*, 472 bp for *Glu-B3c*, 662 bp for *Glu-B3d*, 669 bp for *Glu-B3e*, 853 bp for *Glu-B3g*, 1022 bp for *Glu-B3h*, and 621 bp for *Glu-B3i*, indicating that the *Glu-B3 *alleles could be well differentiated based on corresponding markers. Detection of *Glu-B3f *required the use of the *Glu-B3fg *marker with an 812-bp marker in combination with the *Glu-B3g *marker since no *Glu-B3f *allele-specific marker has been designed. Although *Glu-B3f *could not be clearly distinguished from *Glu-B3g *by protein based methods, these alleles could be definitively differentiated by PCR. In addition, there were obvious differences between genes *GluB3-1 *and *GluB3-2 *in the gene sequences of *Glu-B3f *and *Glu-B3g *[28]. The differences were firstly, the sequence length of *Glu-B3f *was 60 bp longer than that of *Glu-B3g *in the *GluB3-1 *gene, and secondly, there were single base differences between *Glu-B3f *and *Glu-B3g *in both *GluB3-1 *and *GluB3-2*. Therefore, alleles *Glu-B3f *and *Glu-B3g *reported in previous studies were different alleles although they could not be reliably differentiated by SDS-PAGE, 2-DE or MALDI-TOF-MS [13,34].

*Glu-D3 *appeared to be the most complicated locus. It contains the highest number of genes and expressed subunits compared to the other two loci, and yet most of the subunits across different alleles have similar molecular weights. Electrophoresis based methods and PCR are not efficient in differentiating *Glu-D3 *alleles. The MALDI-TOF-MS based method can differentiate *Glu-D3 *alleles since it is able to differentiate subtle changes in mass values. High accuracy mass calibration to remove the variations in mass measurement is the key to improve the efficiency of MALDI-TOF in differentiating these alleles.

### Comparison of the four methods for identification of LMW-GS composition

The data from all five laboratories and the four methods employed showed that alleles *Glu-A3b*, *Glu-A3d *and *Glu-A3e *were consistently identified by all four methods. Similarly, analyses of alleles *Glu-B3a*, *Glu-B3b*, *Glu-B3c*, *Glu-B3h *and *Glu-B3j *were in agreement for all four methods. At the *Glu-D3*, only the *Glu-D3c *allele was consistently identified by SDS-PAGE, 2-DE and MALDI-TOF-MS. The discrepancies in allelic identification using the different methods are indicated in Table 2. Alleles *Glu-A3a *and *Glu-A3c *could not be distinguished by MALDI-TOF-MS due to their nearly identical molecular masses. Similarly, these two alleles could not be reliably identified by SDS-PAGE and 2-DE due to their identical mobilities and pI. However, it was easy to differentiate them by PCR. In SDS-PAGE gels, the higher mobility patterns of alleles *Glu-B3d*, *Glu-B3h*, *Glu-B3i *overlapped with those of alleles *Glu-A3a *or *Glu-A3c*, and lower mobility patterns overlapped with those of allele *Glu-A3b*. These results were in agreement with the reports of Gupta and Shepherd [13], who concluded that ambiguous identification of subunits was possibly caused by differential staining intensity of banding patterns. The difficulty to differentiate *Glu-B3b *and *Glu-B3g *based on SDS-PAGE banding patterns arose from their similar mobilities. However, as shown in Figures 3 and 4, several *Glu-B3 *alleles could be readily discriminated using gliadins as a marker for glutenin by SDS-PAGE. These alleles had clearly different peaks or spots using MALDI-TOF-MS or 2-DE, respectively. Alleles *Glu-D3a *and *Glu-D3b *could not be reliably separated by MALDI-TOF-MS or 2-DE. It is suggested that the *Glu-D3 *alleles should be differentiated by a combination of primers [49-51].

**Table 2:** Allelic variants of LMW-GS identified using different methods

Locus	Subunit	SDS-PAGE	2-DE	MALDI-TOF-MS	PCR
*Glu-A3*	*Glu-A3a*	√*	√	√	√
	*Glu-A3b*	√	√	√	√
	*Glu-A3c*	√	√	√	√
	*Glu-A3d*	√	√	√	√
	*Glu-A3e*	√	√	√	√
	*Glu-A3f*		√	√	√
	*Glu-A3g*		√		√

*Glu-B3*	*Glu-B3a*	√	√	√	√
	*Glu-B3b*	√	√	√	√
	*Glu-B3c*	√	√	√	√
	*Glu-B3d*	√		√	√
	*Glu-B3f*			√	√
	*Glu-B3g*	√	√	√	√
	*Glu-B3h*	√	√	√	√
	*Glu-B3i*	√			√
	*Glu-B3j*	√	√	√	√
	*Glu-B3ab*		√		
	*Glu-B3ac*		√		
	*Glu-B3ad*		√		

*Glu-D3*	*Glu-D3a*	√		√	-
	*Glu-D3b*	√		√	-
	*Glu-D3c*	√	√	√	-
	*Glu-D3m*		√	√	-
	*Glu-D3l*	√	√		-
	*Glu-D3n*		√		

√ - confirmed; - data not available

The 2-DE method is generally considered as the most powerful tool for identifying storage protein polymorphism of proteins in wheat [52]. However, different bands in SDS-PAGE separations were not always distinguishable in 2-DE separations. For example, alleles *Glu-B3d *and *Glu-B3i *could be identified by SDS-PAGE, but not by 2-DE. For LMW-GS identification in wheat breeding programs, PCR and/or SDS-PAGE of both gliadin and glutenin extracts should be used as the basic method, with 2-DE and MALDI-TOF-MS as complementary approaches. A combination of different methods is recommended for differentiating certain LMW-GS alleles, particularly those suspected as being novel.

Comparison of the four methods is presented in Table 3. Utilization of a particular method will depend upon research objectives and the targeted materials. With appropriate classification of glutenin alleles, it is possible to improve wheat quality by selection of alleles and allelic combinations with desired quality performance. If progeny screening and cultivar development is the objective, PCR will likely be adequate for the identification of *Glu-A3 *and *Glu-B3 *alleles. However, if the aim is to determine the glutenin subunits of potential parents for predicting cross performance and designing crossing schemes, or to identify specific alleles such as *Glu-A3g*, *Glu-B3ab*, *Glu-B3ac*, or distinguish between the *Glu-D3 *alleles, a combination of methods should be used, i.e. PCR with 2-DE or PCR with SDS-PAGE and 2-DE, in order to achieve the correct identification of LMW-GS alleles.

**Table 3:** Relative efficiencies of methods of gluten analysis for situations where cultivar identification is required

Subject	SDS-PAGE	2-DE	MALDI-TOF-MS	PCR
Required sample amount	40 μg (Protein)	150 μg (Protein)	0.04 μg (Protein)	2 μL (DNA)
Purity required	Low	High	High	Medium
Number of alleles	19	22	21	16
Alleles efficiently resolved	*Glu-B3b *and *Glu-B3g*, *Glu-B3d *and *Glu-B3i*,	*Glu-A3e*, *Glu-A3f*, *Glu-A3g*, *Glu-B3b*, *Glu-B3g*, *Glu-B3ab*, *Glu-B3ac*, *Glu-B3ad*, *Glu-D3l *and *Glu-D3m*	*Glu-A3e*, and *Glu-A3f; Glu-D3a, Glu-D3b, Glu-D3c, and Glu-D3m*	*Glu-A3e *and *Glu-A3f*, *Glu-B3d *and *Glu-B3i*, *Glu-B3f *and *Glu-B3g*
Mass accuracy	Inaccurate	Inaccurate	Accurate	Accurate
pI	Unknown	Known	Unknown	Unknown
Cost of equipment	≈$7,000	≈$30,000	≈$20,000-400,000	≈$5,500
Cost per sample	≈$1.0	≈$70.0	≈$0.3	≈$0.3
Number of samples analysed per day for skilled technician	30-160*	1	100	100
Automation	Not possible	Not possible	Possible	Possible
Experience required	Considerable	Considerable	Less	Less
Safety	High toxicity	High toxicity	Safe	Toxicity
False positives	No	Yes	No	Yes
Accuracy level	++	+++	++	++

*. Thirty samples/day if running two gels. Up to 160 samples/day if using multi-gel (8 gels) buffer tank.

### A set of standard cultivars for identification of LMW-GS

From this study of 103 wheat cultivars from 12 countries we propose a set of 30 cultivars for determination of LMW-GS (Table 4) irrespective of the method to be used. Figures 1, 3, 5 show glutenin electropherograms of 28 (missing Ernest and Darius) of the 30 genotypes presented in Table 4. They cover all LMW-GS allelic variants identified in the original set. A core set of Chinese Spring, Opata 85, Seri 82 and Pavon 76 is recommended for inclusion in all gels. Most of the common *Glu-*3 alleles are represented among this group and their distributions on gels will provide useful landmarks for comparison with other bands. In this classification, it is possible to differentiate alleles *Glu-A3g *from *Glu-A3d*, *Glu-B3ab *from *Glu-B3b*, *Glu-B3ac *from *Glu-B3g*, *Glu-B3ad *from *Glu-B3i*, and *Glu-D3l *from *Glu-D3c*. Alleles *Glu-D3e *and *Glu-D3c *are assumed to be identical. The allele in cultivar Darius, with no distinct spot in 2-DE gels, is a new allele, *Glu-D3m*. The new allele *Glu-D3n *identified in the cultivar Fengmai 27 has a distinct spot in 2-DE and different mobility in SDS-PAGE (Figure 5). However, more work is needed to further characterize these new alleles at the *Glu-D3 *locus. The other alleles were the same as those observed by Gupta and Shepherd [13].

**Table 4:** Thirty cultivars recommended as standards for the determination of LMW-GS alleles

Locus	Allele	Standard cultivar
*Glu-A3*	*Glu-A3a*	Neixiang 188, **Chinese Spring**
	*Glu-A3b*	Gabo, **Pavon 76**
	*Glu-A3c*	Pitic, **Seri 82**
	*Glu-A3d*	Nidera Baguette 10, Cappelle-Desprez
	*Glu-A3e*	Amadina, Marquis
	*Glu-A3f*	Kitanokaori, Renan
	*Glu-A3g*	Bluesky, Glenlea

*Glu-B3*	*Glu-B3a*	**Chinese Spring**
	*Glu-B3b*	Renan, Gabo
	*Glu-B3c*	Insignia, Halberd
	*Glu-B3d*	Pepital, Ernest
	*Glu-B3f*	Fengmai 27
	*Glu-B3g*	Splendor, Cappelle-Desprez
	*Glu-B3h*	Aca 303, **Pavon 76**
	*Glu-B3i*	Norin 61
	*Glu-B3j*	Grebe, **Seri 82**
	*Glu-B3ab*	Nanbu-komugi
	*Glu-B3ac*	Thesee, Aca 801
	*Glu-B3ad*	Heilo, **Opata 85**

*Glu-D3*	*Glu-D3a*	**Chinese Spring**, Neixiang 188
	*Glu-D3b*	Gabo, Avocet
	*Glu-D3c*	Insignia, Cappelle-Desprez
	*Glu-D3m*	Darius
	*Glu-D3l*	Amadina, Heilo
	*Glu-D3n*	Fengmai 27

The core group is in bold

Allele *Glu-A3g*, identified in the Canadian cultivars Bluesky and Glenlea by 2-DE in the current collection, is widely distributed in many cultivars from Canada and the U.S.A. [41]. In previous studies, allele *Glu-A3g *was frequently identified as *Glu-A3d *due to their similar SDS-PAGE patterns. The role of *Glu-A3g *in bread making quality therefore requires further study. Similarly, effects on bread making quality of alleles *Glu-B3ab*, *Glu-B3ac*, *Glu-B3ad *and *Glu-D3l*, with two additional distinct spots compared to alleles *Glu-B3b*, *Glu-B3g*, *Glu-B3i *and *Glu-D3c*, respectively, also need further investigation.

## Conclusions

Four methods, SDS-PAGE, 2-DE, MALDI-TOF-MS and PCR, were used for identifying the LMW-GS composition in wheat cultivars from 12 countries. All seven *Glu-A3 *alleles could be identified by 2-DE and PCR, and only four and five of the seven could be differentiated by MALDI-TOF-MS and SDS-PAGE of the glutenin extract, respectively. The *Glu-B3 *alleles *Glu-B3a*, *Glu-B3b*, *Glu-B3c*, *Glu-B3g*, *Glu-B3h *and *Glu-B3j *could be identified by all four methods, but alleles *Glu-B3ab*, *Glu-B3ac*, *Glu-B3ad *could only be identified by the 2-DE method. *Glu-D3 *alleles were very difficult to clearly distinguish by SDS-PAGE, 2-DE and PCR. MALDI-TOF-MS was promising in reliably differentiating them. PCR is a simple, accurate, and low cost method for identifying *Glu-A3 *and *Glu-B3 *alleles that are currently routinely analysed by SDS-PAGE in breeding programs. However, SDS-PAGE using a multi-gel buffer chamber, and running both gliadins and glutenin extracts is also a highly reliable method. A combination of all methods will help to identify specific alleles, especially potentially new alleles.

A set of 30 cultivars (Table 4) was recommended for identifying LMW-GS alleles. These standard cultivars cover all variants of LMW-GS in the collection investigated. Among them, Chinese Spring, Opata 85, Seri 82 and Pavon 76, are recommended as a core set to be included in each SDS-PAGE gel when identifying alleles of LMW-GS genes. The 30 cultivars have been placed in CIMMYT's and INRA Clermont Ferrand, France germplasm banks and seed is being multiplied to make them freely available as a set upon request. Accession numbers will be assigned once the *Glu-1/Glu-3 *allelic composition is confirmed.

## Methods

### Plant materials

One hundred and three cultivars of common wheat collected from 12 countries were used to develop a set of standard cultivars for identification of LMW-GS alleles (Table 1). They included 21 cultivars from China, 19 from Argentina, 15 from Australia, 14 from France, 10 from Japan, eight from Mexico, seven from Canada, three from the USA, two from Italy, two from the Netherlands, one from Finland and one from Germany. These cultivars were widely utilized in investigating glutenin subunit compositions and their relationships to processing quality [41].

### Protein extraction

A similar protocol was adopted for protein extraction in all five laboratories. Proteins were extracted from 100 mg whole meal according to the sequential procedure of Branlard and Bancel [53]. The samples were treated with 1.0 mL of 50% propanol-1-ol (v/v) for 5 min with continuous vortexing, followed by incubation (20 min at 65°C), vortexing (5 min), and centrifugation (5 min at 10, 000 × g). This step was repeated three times to remove most of the gliadins. The glutenin in the pellet was reduced with 50% propanol-1-ol, 50 mM Tris-HCl solution containing 1% w/v dithiothreitol (DTT), after which 1.4% v/v of 4-vinylpyridine was added, and alkylation was continued overnight at room temperature. The protein of each cultivar was extracted in three replicates.

### SDS-PAGE

SDS-PAGE was performed in all five laboratories. Glutenin and gliadin protein extracts were separated using the method of Singh et al. [46] with some modifications in different laboratories to obtain the best resolution. To summarize, there were differences in three aspects. The concentrations of separation gel were 14.0% concentration (T) with 1.3% cross linker (C), 15.0% T with 1.3% C, 12.5% T with 0.97% C, 15.0% T with 1.4% C, and 13.5% T with 0.8% C in the laboratory of CAAS, CIMMYT, INRA, NARO and Universidad Nacionalm of Argentina, respectively. The pH for separation gel was pH8.8 in all laboratories except in CIMMYT with pH8.5. The currents of running gel were 16, 12.5, 30, 30 and 40 mA in the laboratory of CAAS, CIMMYT, INRA, NARO and Universidad Nacionalm of Argentina laboratory, respectively. Generally, lower current results in better resolution, but we could not find the optimum conditions for maximum resolution of LMW-GS in all laboratories since each laboratory used its own optimum conditions. Details were reported by Ikeda et al. [35].

The LMW-GS compositions were identified according to Singh et al. [46] and Jackson et al. [16] and the gliadins were used as indicators of LMW-GS based on the linkage between LMW-GS and gliadin because the gliadin composition can be screened more readily than specific LMW-GS. The nomenclature system of LMW-GS followed Gupta and Shepherd [13], Jackson et al. [16], Branlard et al. [34], Ikeda et al. [35], Appelbee et al. [19] and the catalogue of gene symbols for wheat http://wheat.pw.usda.gov/ggpages/awn/53/Textfile/WGC.html.

### 2-DE procedure

The 2-DE method was only performed at CAAS and NARO. The 2-DE procedure employed to identify LMW-GS was performed with an IPGphor (GE Healthcare, Sweden) for isoelectric focusing (IEF), and an AE-6530 chamber and an AE-8450 power supply (ATTO, Japan) for SDS-PAGE. The glutenin fraction was precipitated with 80% acetone [54], and the resulting pellets containing 150 μg protein were dissolved in 250 μL of IEF rehydration solution [7 M urea, 2 M thiourea, 4% w/v CHAPS, 2% v/v IPG buffer pH 6-11 (GE Healthcare) and 20 mM DTT] for very basic proteins [55]. After incubation for 30 min at room temperature, samples were applied to Immobiline Dry-Strip pH 6-11 (13 cm, GE Healthcare). The rehydration step was carried out for 12 h at 20°C. IEF was performed with a step-wise protocol to 45 kVh. After IEF, the strips were stored at -80°C or prepared directly for 2-DE as follows: the gel strips were first equilibrated under gentle shaking for 15 min in equilibration buffer (50 mM Tris-HCl, pH 8.8, 6 M urea, 30% v/v glycerol, 2% w/v SDS) with 2% w/v DTT, and then in equilibration buffer containing 1.4% v/v 4-vinylpyridine. The second dimension separations (SDS-PAGE) were carried out on 13% acrylamide constant gels and ran at 7 mA/gel for 45 min and then 25 mA/gel for approximately 4 h, until the bromophenol blue had run off the bottom of the gel [56]. After the completion of 2-DE, gels were fixed and stained with Coomassie Brilliant Blue-G250 according to Neuhoff et al. [57]. The resulting gels were scanned using an Image Scanner (GE Healthcare) and the images analyzed with ImageMaster 2D Platinum v6.0 software (GE Healthcare). At least three gel images of each sample were taken and compared. The LMW-GS compositions were identified with the distinctive spot on 2-DE gels according to Ikeda et al. [18]. The nomenclature system of LMW-GS was the same as above SDS-PAGE separation.

In some cases the 2-DE was modified where glutenin proteins were not alkylated; 16% isopropanol was added to the IEF buffer, and IEF was performed at 18 kVh [18].

### MALDI-TOF-MS protocol

MALDI-TOF-MS was performed at the State Agriculture Biotechnology Center, Murdoch University, Australia. The glutenin fraction was precipitated with 80% acetone [54], and the resulting pellets containing 100 μg protein were dissolved in 60 μL acetonitrile (ACN)/H_2_O (50:50 v/v) containing 0.05% v/v trifluoroacetic acid (TFA) for 1 h at room temperature. Sample preparation was carried out according to the dried droplet method [58], using sinapinic acid (SA) as matrix. The matrix solution was prepared by dissolving SA in 50% ACN/0.05% TFA (w/w) at a concentration of 10 mg/mL. A sandwich matrix/sample/matrix 1:1:1 (0.7 μL) was deposited on to a 96-sample MALDI target, and dried at room temperature.

MALDI-TOF-MS was performed on a Voyager DE-PRO TOF mass spectrometer (Applied Biosystems, Foster City, CA, USA) equipped with a 337 nm nitrogen laser and delayed extraction. Analyses were carried out on a positive linear ion mode at a mass range of 10000-50000 m/z with an accelerating voltage of 25 kV and a delay time of 900 ns. A low mass gate value of 10000 m/z was selected for analysis to avoid saturation of the detector. The identification of LMW-GS alleles based on MALDI-TOF-MS was established using a set of 19 near-isogenic lines (NIL) of cultivar Aroona (unpublished data, A Wang, W Ma, R Appels, Murdoch University, Australia).

### DNA extraction and PCR amplification

PCR was performed only at CAAS. Genomic DNA was extracted from seeds using a modified CTAB procedure [59]. PCR was performed using TaKaRa (Dalian, China) *Taq *DNA polymerase (1.0 unit) in 20 μL reaction volumes containing approximately 50 ng of genomic DNA, 1× PCR buffer (1.5 mM MgCl_2_), 100 μM of each dNTP and 7.5 pmol of each PCR primer. Details of allele-specific markers for the discrimination of *Glu-A3 *and *Glu-B3 *alleles and PCR conditions were reported previously [27,28].

## Abbreviations

2-DE: two-dimensional gel electrophoresis (IEF × SDS-PAGE); ACN: acetonitrile; BIOLAB AZUL: Laboratory of Functional Biology and Biotechnology; CAAS: Chinese Academy of Agricultural Sciences; CEBB-MdP: Biotechnology and Biodiversity Study Center, Mar del Plata, Argentina; CHAPS: 3-[(3-Cholanidopropyl) dimethylammonio]-1-propanesulfonate; CICPBA: Scientific Research Commission of the Province of Buenos Aires; CIMMYT:International Maize and Wheat Improvement Center; CIISAS: Center for Integrated Research in Sustainable Agricultural Systems, COUNTRY; CONICET: National Science and Technology Research Council, Argentina; CRESCAA: Regional Center for Systemic Study of Agro-alimentary Chains, COUNTRY; CTAB: cetyltrimethylammonium bromide; DNA: deoxyribonucleic acid; dNTPs: deoxynucleoside triphosphates; DTT: dithiothreitol; HMW-GS: high-molecular-weight glutenin subunits; HPLC: high-performance liquid chromatography; IEF: isoelectric focusing; INBA: Research Institute of Agricultural and Environmental Biosciences; INRA: National Institute for Agricultural Research; kDa: kilodalton; kVh: kilo-volt-ampere-hour; LMW-GS: low-molecular-weight glutenin subunits; MALDI-TOF-MS: matrix-assisted laser desorption/ionization time-of-flight mass spectrometry; MgCl_2_: magnesium chloride; NARO: National Agriculture and Food Research Organization; NIL: near-isogenic lines; PCR: polymerase chain reaction; pI: isoelectric points; SA: sinapinic acid; SDS: sodium dodecyl sulfate; SDS-PAGE: sodium dodecyl sulphate-polyacrylamide gel electrophoresis; TFA: trifluoroacetic acid; Tris-HCl: tris (hydroxymethyl) aminomethane hydrochloride

## Authors' contributions

LL carried out SDS-PAGE, 2-DE and MALDI-TOF-MS analyses and drafted the manuscript. TI participated in the design of the study, and performed SDS-PAGE and 2-DE analyses. GB participated in the design of the study and carried out SDS-PAGE analysis. RP participated in the design of the study and carried out the identification of LMW-GS by SDS-PAGE. WR participated in the design of the study and revised the manuscript. SL carried out SDS-PAGE analysis and revised the manuscript. MK carried out SDS-PAGE analysis. XX participated in the design of the study and revised the manuscript. LW carried out PCR analysis. WM participated in the identification of LMW-GS by MALDI-TOF-MS. RA participated in the identification of LMW-GS by MALDI-TOF-MS and revised the manuscript. HY participated in the design of the study. AW participated the identification of LMW-GS by MALDI-TOF-MS. YY participated the identification of LMW-GS by 2-DE. ZH conceived of the study and participated in its design, coordinated the various research groups, and revised the manuscript. All authors read and approved the final manuscript.

## References

[B1] Payne PI Genetics of wheat storage protein and the effect of allelic variation on pan bread quality Annu Rev Plant Physiol 1987 38 141 153 10.1146/annurev.pp.38.060187.001041

[B2] Gianibelli MC Larroque OR MacRichie F Wrigley CW Biochemical, genetic, and molecular characterization of wheat glutenin and its component subunits Cereal Chem 2001 78 6 635 646 10.1094/CCHEM.2001.78.6.635

[B3] Shewry PR Halford NG Lafiandra D Genetics of wheat gluten proteins Adv Genet 2003 49 111 184 full_text 12779252 10.1016/s0065-2660(03)01003-4

[B4] Shewry PR Tatham AS The prolamin storage proteins of cereal seeds, structure and evolution Biochem J 1990 267 1 1 12 2183790 10.1042/bj2670001 PMC1131235

[B5] Bietz JA Wall JS Isolation and characterization of gliadin-like subunits from glutelin Cereal Chem 1973 50 5 537 547

[B6] Cornish GB Békés F Allen HM Martin JM Flour proteins linked to quality traits in an Australian doubled haploid wheat population Aust J Agric Res 2001 52 12 1339 1348 10.1071/AR01060

[B7] Gupta RB Paul JG Cornish GB Palmer GA Bekes F Rathjen AJ Allelic variation at glutenin subunit and gliadin loci, *Glu-1*, *Glu-3 *and *Gli-1*, of common wheats. I. Its additive and interaction effects on dough properties J Cereal Sci 1994 19 1 9 17 10.1006/jcrs.1994.1003

[B8] He ZH Liu L Xia XC Liu JJ Peña RJ Composition of HMW and LMW glutenin subunits and their effects on dough properties, pan bread, and noodle quality of Chinese bread wheats Cereal Chem 2005 82 4 345 350 10.1094/CC-82-0345

[B9] Békés F Morell M Appels R, Eastwood R, Lagudah E, Langridge P, Mackay M, McIntyre L, Sharp P An integrated approach to predicting end-product quality of wheat Proc 11th Int Wheat Genet Symp, Sydney University Press, Sydney, Australia 2008 O45

[B10] Singh NK Shepherd FW Linkage mapping of genes controlling endosperm storage proteins in wheat. 1. Genes on the short arms of group 1 chromosomes Theor Appl Genet 1988 75 4 628 641 10.1007/BF00289132

[B11] Pogna NE Autran JC Mellini F Lafiandra D Feillet P Chromosome 1B-encoded gliadins and glutenin subunits in durum wheat, genetics and relationship to gluten strength J Cereal Sci 1990 11 1 15 34 10.1016/S0733-5210(09)80178-1

[B12] Anderson OD Gu YQ Kong XY Lazo GR Wu JJ The wheat ω-gliadin genes: structure and EST analysis Funct Integr Genomics 2009 9 3 397 410 10.1007/s10142-009-0122-2 19367421 PMC2700870

[B13] Gupta RB Shepherd KW Two-step one-dimensional SDS-PAGE analysis of LMW subunits of glutelin. 1. Variation and genetic control of the subunits in hexaploid wheats Theor Appl Genet 1990 80 1 65 74 10.1007/BF00224017 24220812

[B14] Yan Y Hsam SL Yu JZ Jiang Y Ohtsuka I Zeller FJ HMW and LMW glutenin alleles among putative tetraploid and hexaploid European spelt wheat (*Triticum spelta *L.) progenitors Theor Appl Genet 2003 107 7 1321 1330 10.1007/s00122-003-1315-z 13679994

[B15] Lerner SE Kolman MA Rogers WJ Quality and endosperm storage protein variation in Argentinean grown bread wheat. I. Allelic diversity and discrimination between cultivars J Cereal Sci 2009 49 3 337 345 10.1016/j.jcs.2008.04.003

[B16] Jackson EA Morel MH Sontag-Strohm T Branlard G Metakovsky EV Redaelli R Proposal for combining the classification systems of alleles of *Gli-1 *and *Glu-3 *loci in bread wheat (*Triticum aestivum *L.) J Genet Breed 1996 50 321 336

[B17] Eagles HA Hollamby GJ Gororo NN Eastwood RF Estimation and utilisation of glutenin gene effects from the analysis of unbalanced data from wheat breeding programs Aust J Agric Res 2002 53 4 367 377 10.1071/AR01074

[B18] Ikeda TM Araki E Fujita Y Yano H Characterization of low-molecular-weight glutenin subunit genes and their protein products in common wheats Theor Appl Genet 2006 112 2 327 334 10.1007/s00122-005-0131-z 16283233

[B19] Appelbee MJ Mekuria GT Nagasandra V Bonneau JP Eagles HA Eastwood RF Mather DE Novel allelic variants encoded at the *Glu-D3 *locus in bread wheat J Cereal Sci 2009 49 2 254 261 10.1016/j.jcs.2008.10.011

[B20] D'Ovidio R Masci S The low-molecular-weight glutenin subunits of wheat gluten J Cereal Sci 2004 39 3 321 339 10.1016/j.jcs.2003.12.002

[B21] Anderson NG Tollaksen SL Pascoe FH Anderson L Two-dimensional electrophoretic analysis of wheat seed proteins Crop Sci 1985 25 4 667 674

[B22] Dworschak RG Ens W Standing KG Preston KR Marchylo BA Nightingale MJ Stevenson SG Hatcher DW Analysis of wheat gluten proteins by matrix-assisted laser desorption/ionization mass spectrometry J Mass Spectrom 1998 33 5 429 435 10.1002/(SICI)1096-9888(199805)33:5<429::AID-JMS645>3.0.CO;2-O

[B23] Ghirardo A Sørensen HA Petersen M Jacobsen S Søndergaard I Early prediction of wheat quality, analysis during grain development using mass spectrometry and multivariate data analysis Rapid Commun Mass Spectrom 2005 19 4 525 532 10.1002/rcm.1823 15655793

[B24] Muccilli V Cunsolo V Saletti R Foti S Masci S Lafiandra D Characterization of B- and C-type low molecular weight glutenin subunits by electrospray ionization mass spectrometry and matrix-assisted laser desorption/ionization mass spectrometry Proteomics 2005 5 3 719 728 10.1002/pmic.200401029 15682464

[B25] Liu L Wang AL Appels R Ma JH Xia XC Lan P He ZH Bekes F Yan YM Ma WJ A MALDI-TOF based analysis of high molecular weight glutenin subunits for wheat breeding J Cereal Sci 2009 50 2 295 301 10.1016/j.jcs.2009.05.006

[B26] Zhang W Gianibelli MC Rampling LR Gale KR Characterisation and marker development for low molecular weight glutenin genes from *Glu-A3 *alleles of bread wheat (*Triticum aestivum *L.) Theor Appl Genet 2004 108 7 1409 1419 10.1007/s00122-003-1558-8 14727031

[B27] Wang LH Li GY Peña RJ Xia XC He ZH Development of STS markers and establishment of multiplex PCR for *Glu-A3 *alleles in common wheat (*Triticum aestivum *L.) J Cereal Sci 2010 51 3 305 312 10.1016/j.jcs.2010.01.005

[B28] Wang LH Zhao XL He ZH Ma W Appels R Peña RJ Xia XC Characterization of low-molecular-weight glutenin subunit *Glu-B3 *genes and development of STS markers in common wheat (*Triticum aestivum *L.) Theor Appl Genet 2009 118 3 525 539 10.1007/s00122-008-0918-9 18989655

[B29] Beckwith AC Nielsen HC Wall JS Huebner FR Isolation and characterization of a high-molecular-weight protein from wheat gliadin Cereal Chem 1966 43 1 14 28

[B30] Elton GAH Ewart JAD Glutenins and gliadins electrophoretic studies J Sci Food Agric 1966 17 1 34 38 10.1002/jsfa.2740170108

[B31] Jackson EA Holt LM Payne PI Characterisation of high molecular weight gliadin and low-molecular-weight glutenin subunits of wheat endosperm by two-dimensional electrophoresis and the chromosomal localisation of their controlling genes Theor Appl Genet 1983 66 1 29 37 10.1007/BF00281844 24263628

[B32] Ikeda TM Nagamine T Fukuoka H Yano H Identification of new low-molecular-weight glutenin subunit genes in wheat Theor Appl Genet 2002 104 4 680 687 10.1007/s001220100756 12582674

[B33] Juhász A Gianibelli MC Lafiandra D, Masci S, D'Ovidio R Information hidden in the low molecular weight glutenin gene sequences Proc 8th Gluten Workshop, Bitervo, Italy, The Gluten Proteins 2003 62 65

[B34] Branlard G Dardevet M Amiour N Igrejas G Allelic diversity of HMW and LMW glutenin subunits and omega-gliadins in French bread wheat (*Triticum aestivum *L.) Genet Resour Crop Evol 2003 50 7 669 679 10.1023/A:1025077005401

[B35] Ikeda TM Branlard G Peña RJ Takata K Liu L He ZH Lerner SE Kolman MA Yoshida H Rogers WJ Appels R, Eastwood R, Lagudah E, Langridge P, Mackay M, McIntyre L, Sharp P International collaboration for unifying *Glu-3 *nomenclature systems in common wheat Proc 11th Int Wheat Genet Symp, Sydney University Press, Sydney, Australia 2008 O42

[B36] Lew EJL Kuzmicky DD Kasarda DD Characterization of low molecular weight glutenin subunits by reversed-phase high performance liquid chromatography, sodium dodecyl sulphate-polyacrylamide gel electrophoresis, and N-terminal amino acid sequencing Cereal Chem 1992 69 5 508 515

[B37] Tao HP Kasarda DD Two-dimensional gel mapping and N-terminal sequencing of LMW-glutenin subunits J Exp Bot 1989 40 9 1015 1020 10.1093/jxb/40.9.1015

[B38] Masci S Rovelli L Kasarda DD Vensel WH Lafiandra D Characterisation and chromosomal localization of C-type low-molecular-weight glutenin subunits in the bread wheat cultivar Chinese Spring Theor Appl Genet 2002 104 2 422 428 10.1007/s001220100761 12582715

[B39] Masci S Lafiandra D Porceddu E Lew EJL Tao HP Kasarda DD D-glutenin subunits, N-terminal sequences and evidence for the presence of cysteine Cereal Chem 1993 70 5 581 585

[B40] Nieto-Taladriz MT Rodriguez-Quijano M Carrillo JM Biochemical and genetic characterization of a D glutenin subunit encoded at the *Glu-B3 *locus Genome 1998 41 2 215 220 10.1139/gen-41-2-215

[B41] Wrigley CW Bekes F Bushuk W The gluten composition of wheat varieties and genotypes AACC International 2006 ISBN 1-891127-51-9

[B42] Cornish GB Burridge PM Palmer GA Wrigley CW Wrigley CW Mapping the origins of some HMW and LMW glutenin subunit alleles in Australian germplasm Proc 43rd Aust Cereal Chem Conf, Royal Aust Chem Inst, Melbourne 1993 255 260

[B43] Branlard G Dardevet R Saccomano F Lagoutte F Gourdon J Genetic diversity of wheat storage proteins and bread wheat quality Euphytica 2001 119 1 59 67 10.1023/A:1017586220359

[B44] Luo C Griffin WB Branlard G McNeil DL Comparison of low and high molecular weight wheat glutenin allele effects on flour quality Theor Appl Genet 2001 102 6 1088 1098 10.1007/s001220000433

[B45] Vawser MJ Cornish GB Shepherd KW Black CK, Panozzo JF, Wrigley CW, Batey IL, Larsen N Rheological dough properties of Aroona isolines differing in glutenin subunit composition Proc 52nd Aust Cereal Chem Conf, Royal Aust Chem Inst, Christchurch, New Zealand 2002 53 58

[B46] Singh NK Shepherd KW Cornish GB A simplified SDS-PAGE procedure for separating LMW subunits of glutenin J Cereal Sci 1991 14 3 203 208 10.1016/S0733-5210(09)80039-8

[B47] Liu L He ZH Yan J Zhang Y Xia XC Peña RJ Allelic variation at the *Glu-1 *and *Glu-3 *loci, presence of the 1B/1R translocation, and their effects on mixographic properties in Chinese bread wheats Euphytica 2005 142 3 197 204 10.1007/s10681-005-1682-4

[B48] Marchylo BA Handel KA Mellish VJ Fast horizontal sodium dodecyl sulfate gradient polyacrylamide gel electrophoresis for rapid wheat cultivar identification and analysis of high molecular weight glutenin subunits Cereal Chem 1989 66 3 186 192

[B49] Zhao XL Ma W Gale KR Lei ZS He ZH Sun QX Xia XC Identification of SNPs and development functional markers for LMW-GS genes at *Glu-D3 *and *Glu-B3 *loci in bread wheat (*Triticum aestivum *L.) Mol Breed 2007 20 3 223 231 10.1007/s11032-007-9085-y

[B50] Zhao XL Xia XC He ZH Gale KR Lei ZS Appels R Ma WJ Characterization of three low-molecular-weight *Glu-D3 *subunit genes in common wheat Theor Appl Genet 2006 113 7 1247 1259 10.1007/s00122-006-0379-y 16941095

[B51] Zhao XL Xia XC He ZH Lei ZS Appels R Yang Y Sun QX Ma W Novel DNA variations to characterize low molecular weight glutenin *Glu-D3 *genes and develop STS markers in common wheat Theor Appl Genet 2007 114 3 451 460 10.1007/s00122-006-0445-5 17106734

[B52] Yahata E Maruyama-Funatsuki W Nishio Z Tabiki T Takata K Yamamoto Y Tanida M Saruyama H Wheat cultivar-specific proteins in grain revealed by 2-DE and their application to cultivar identification of flour Proteomics 2005 5 15 3942 3953 10.1002/pmic.200402103 16152659

[B53] Branlard G Bancel E Grain protein extraction. Plant proteomics, methods and protocols Methods Mol Biol 2006 355 15 25 10.1385/1-59745-227-0:15 17093298

[B54] Melas V Morel MH Autran JC Feillet P Simple and rapid method for purifying low molecular weight subunits of glutenin from wheat Cereal Chem 1994 71 3 234 237

[B55] Dumur J Jahier J Bancel E Laurière M Bernard M Branlard G Proteomic analysis of aneuploid lines in the homeologous group 1 of the hexaploid wheat cultivar Courtot Proteomics 2004 4 9 2685 2695 10.1002/pmic.200300800 15352243

[B56] Görg A Obermaier C Boguth G Harder A Scheibe B Wildgruber R Weiss W The current state of two-dimensional electrophoresis with immobilised pH gradients Electrophoresis 2000 21 6 1037 1053 10.1002/(SICI)1522-2683(20000401)21:6<1037::AID-ELPS1037>3.0.CO;2-V 10786879

[B57] Neuhoff V Arold N Taube D Ehrhardt W Improved staining of proteins in polyacrylamide gels including isoelectric focusing gels with clear background at nanogram sensitivity using Coomassie Brilliant Blue G-250 and R-250 Electrophoresis 1988 9 6 255 262 10.1002/elps.1150090603 2466658

[B58] Kussmann M Nordhoff E Rahbek-Nielsen H Haebel S Rossel-Larsen M Jakobsen L Gobom J Mirgorodskaya E Kroll-Kristensen A Palm L Roepstorff P MALDI-MS sample preparation techniques designed for various peptide and protein analytes J Mass Spectrom 1997 32 6 593 601 10.1002/(SICI)1096-9888(199706)32:6<593::AID-JMS511>3.0.CO;2-D

[B59] Gale KR Ma W Zhang W Rampling L Hill AS Appels R Morris P Morell M Eastwood R Simple high-throughput DNA markers for genotyping in wheat Proc 10th Australian Wheat Breeding Assembly, Wheat Breeding Soc of Australia 2001 26 31

